# Targeted next-generation sequencing-based detection of microsatellite instability in colorectal carcinomas

**DOI:** 10.1371/journal.pone.0246356

**Published:** 2021-02-01

**Authors:** Yunbeom Lee, Ji Ae Lee, Hye Eun Park, Hyojun Han, Yuhnam Kim, Jeong Mo Bae, Jung Ho Kim, Nam-Yun Cho, Hwang-Phill Kim, Tae-You Kim, Gyeong Hoon Kang

**Affiliations:** 1 Celemics, Inc. Seoul, Korea; 2 Laboratory of Epigenetics, Cancer Research Institute, Seoul National University College of Medicine, Seoul, Korea; 3 Department of Pathology, Seoul National University College of Medicine, Seoul, Korea; 4 Department of Pathology, Seoul National University Hospital, Seoul, Korea; 5 Laboratory of Cancer Epigenetics, Cancer Research Institute, Seoul National University College of Medicine, Seoul, Korea; 6 Department of Internal Medicine, Seoul National University College of Medicine, Seoul, Korea; CNR, ITALY

## Abstract

In the present study, we developed a computational method and panel markers to assess microsatellite instability (MSI) using a targeted next-generation sequencing (NGS) platform and compared the performance of our computational method, mSILICO, with that of mSINGS to detect MSI in CRCs. We evaluated 13 CRC cell lines, 84 fresh and 119 formalin-fixed CRC tissues (including 61 MSI-high CRCs and 155 microsatellite-stable CRCs) and tested the classification performance of the two methods on 23, 230, and 3,154 microsatellite markers. For the fresh tissue and cell line samples, mSILICO showed a sensitivity of 100% and a specificity of 100%, regardless of the number of panel markers, whereas for the formalin-fixed tissue samples, mSILICO exhibited a sensitivity of up to 100% and a specificity of up to 100% with three differently sized panels ranging from 23 to 3154. These results were similar to those of mSINGS. With the application of mSILICO, the small panel of 23 markers had a sensitivity of ≥95% and a specificity of 100% in cell lines/fresh tissues and formalin-fixed tissues of CRC. In conclusion, we developed a new computational method and microsatellite marker panels for the determination of MSI that does not require paired normal tissues. A small panel could be integrated into the targeted NGS panel for the concurrent analysis of single nucleotide variations and MSI.

## Introduction

Microsatellites, also known as short tandem repeats, are tracts of tandemly repeated DNA motifs that range from 1 to 6 bp and are typically repeated 10–60 times. Microsatellites are encountered throughout the human genome at a frequency of one microsatellite locus per 2,000 bp, accounting for 3% of the human genome [1]. Approximately 8% of microsatellites are found in coding regions [2]. and the most common microsatellites of coding regions are A homopolymers [3]. Microsatellites tend to undergo slipped strand mispairing during DNA replication, which is repaired by mismatch repair (MMR) enzymes. Microsatellite instability (MSI) is characterized by genome-wide alterations in the number of repeated DNA bases in microsatellites due to defective DNA MMR. High rates of repeat number alterations and increased rates of single nucleotide variations feature MSI-high tumor cells [4]. The MSI-high molecular phenotype is found in colorectal cancers (CRCs) at a frequency of 8–15% and correlates with morphological phenotype [5].

MSI testing of DNA samples obtained from paired tumor and normal tissues is traditionally performed using the National Cancer Institute (NCI)’s five-marker panel (BAT25, BAT26, D2S123, D5S346, and D17S250). MSI status is classified as MSI-high (MSI-H; instability at 2 or more markers) and microsatellite-stable (MSS; instability at 1 marker or none). Recent data suggest that the use of mononucleotide repeats increases the sensitivity of the detection of MSI compared to the use of dinucleotide repeats [6–8]. The revised Bethesda guideline recommended the use of a new panel, the pentaplex panel of five mononucleotide repeats for the detection of MSI [9]. There has been a growing demand to develop more sensitive solutions for the detection of MSI with a larger number of microsatellite loci. Next-generation sequencing (NGS) allows for the analysis of a greater number of microsatellite markers than PCR-based detection of MSI (PCR-MSI). Furthermore, simultaneous analysis of both MSI and SNVs or indels is possible from a single assay of NGS.

NGS-based assessment of MSI (NGS-MSI) has pursued two approaches based on the type of interrogated microsatellite markers, including one approach using microsatellite loci located within the captured gene sequences [10] and the other approach capturing dedicated specific microsatellite marker sites that are not included in targeted gene capture sequencing data. Several computational methods for NGS-MSI have been developed, including MSISensor, mSINGS, MANTIS, and Cortes-Ciriano, which are based on the comparison of the repeat length distribution of microsatellites [11]. Of these computational methods, mSINGS does not need paired normal tissue samples for the detection of MSI in tumor samples [11]. Instead, mSINGS compares tumor-only samples to a pre-constructed baseline-control. In the present study, we developed a new computational method, mSILICO, which is run on tumor samples without matched normal tissue samples. We designed 3,154 capture probes for dedicated microsatellite sites. mSINGS and mSILICO were compared regarding the sensitivity and specificity for the detection of MSI in CRCs.

## Materials and methods

This study was approved by the Institutional Review of Board of Seoul National University Hospital (IRB No. H-1605-080-761). The written informed consent was obtained from CRC patients prior to participating in the present study. This study was conducted in compliance with the principles of the Declaration of Helsinki and its later amendments. Fresh tissue samples of paired CRC tumors and adjacent mucosa were obtained from patients (n = 84) who underwent surgical resection due to CRC in Seoul National University Hospital in 2018. Genomic DNA was extracted from the paired tissue samples using a Qiagen kit (Qiagen, Hilden, Germany). After review of electronic medical records from CRC patients who underwent surgery in 2018, 40 cases of MSI-high CRC and 79 cases of non-MSI-high CRC were selected. After microscopic examination of glass slides, a 1 cm-sized tumor area with the highest tumor purity and a normal tissue area were marked. The corresponding areas were marked on the unstained recut slides and then subjected to deparaffinization. The marked tumor and normal tissue areas were separately scraped into microcentrifuge tubes with a knife blade. Genomic DNA was extracted using the Qiagen FFPE Kit. Cell lines (n = 13) were purchased from Korean Cell Line Bank (Seoul, Korea), and genomic DNA was extracted from the cell lines after culture. The cells were cultured in a 5% CO_2_ humidified atmosphere at 37°C using DMEM (Thermo Fisher Scientific, Waltham, MA, USA) supplemented with 10% fetal bovine serum, 100 μg/mL penicillin and 100 μg/mL streptomycin.

### PCR-MSI using Bethesda’s five-marker panel

Genomic DNA samples obtained from paired tumor and normal tissue samples were subjected to PCR with fluorescently labeled oligonucleotide primers for Bethesda’s five microsatellite loci (BAT25, BAT26, D2S123, D5S346, and D17S250), and then the PCR products were analyzed by capillary electrophoresis on an ABI 3100 Genetic Analyzer (Applied Biosystems, Foster City, CA). Instability at the examined locus was defined by altered length of the PCR product in the tumor sample compared with the length of the PCR product in the paired normal sample. MSI status was classified as follows: MSI-H (instability at 2 or more microsatellite loci) and MSS (instability at 1 locus or none).

### Immunohistochemistry for MMR proteins

Immunohistochemistry was performed to evaluate the expression of the MLH1, MSH2, MSH6, and PMS2 proteins as described previously [12]. The criteria for the microscopic interpretation of each IHC marker were based on our previous study [12].

### Library preparation and next-generation sequencing

The NGS panel was designed for simultaneous detection of MSI status and mutations in 50 CRC-related genes, including 40 genes associated with the WNT, p53, RTK-RAS, TGF-β, and PI3K pathways [13] (S1 Table). Custom RNA probes were designed for target enrichment sequencing (Celemics Inc, Seoul, Korea) and covered all unions of reported exons of 50 genes (total count of regions was 753). The panel covers 777,937 bases of the human genome (hg19). All transcripts of genes reported in UCSC were included as targets to thoroughly detect SNVs, small insertion-deletion mutations, and structural variants. In addition, the panel contains special RNA probes that capture dedicated specific microsatellite marker sites (n = 3,154) for the detection of MSI.

Genomic DNA was sheared and processed for Illumina sequencing. The process included the following steps: end repair, dA tailing, adaptor ligation and pre-PCR for the indexed NGS library. Capture probes were hybridized in buffer to capture target regions of the C5 gene through the use of the Celemics Target Enrichment Kit. After the capture and washing processes were completed, the captured library was amplified by post-PCR. The PCR products were sequenced on the NextSeq 500 platform by Illumina Inc.

‘BCL2FASTQ’ version 2.19.1.403 (Illumina) was used to demultiplex the base-call image files into individual sequence read files (FASTQ format). All options and parameters followed the default setting. Sequencing adaptors were removed by “AdapterRemoval ver. 2.2.2” [14], after low quality bases (below quality 0 and N sequence) were removed by native python code. All sequencing reads were aligned to the hg19 human genome by BWA-MEM (Burrows-Wheeler Aligner) software. The program used the Burrows-Wheeler Transform algorithm to index the human genome sequence to calculate the constant complexity of each sequencing read. Post-align and recalibration processes were performed by ‘Picard’ ver. 1.115 (http://broadinstitute.github.io/picard) and ‘GenomeAnalysisToolKit (GATK ver. 4.0.4.0)’ [15]. We performed variant calling with a GATK haplotype caller. All detailed parameters and options followed GATK best practices.

### MSI marker panels

To select microsatellite markers, we went through a series of processes. First, we referred to previously reported studies that conducted NGS-based determination of MSI [16–19] and retrieved 3,154 marker candidates. Next, to construct an intermediate-sized MSI marker panel, we randomly selected 230 markers from 3,154 markers. Finally, we looked for overlap of the marker candidates across the studies [17, 20], which revealed 18 shared markers. In addition to these 18 markers, three Bethesda markers and two markers from Salipante et al.’s study [16] were added, resulting in 23 markers (S2 Table). Because our NGS panel was designed for simultaneous detection of MSI status and mutations in 50 CRC-related genes, two markers of the Salipante’s study are already present on the genomic sequence covered by the 50-gene panel. Thus, we included these two markers in the 23-marker panel.

### Computational methods for the determination of MSI by NGS

Instability at a specific microsatellite marker was determined using calculation formula which measures the skewness in the distribution of read lengths on a specific microsatellite marker. We collected all the reads mapped on each microsatellite marker and counted the length of each read. The read length was normalized by reference genome (hg19) length, which is set to 0 if the read length is the same as that of the reference. Markers with a sequence coverage (≤20x) were filtered out and excluded from the following analysis. Pearson’s skewness coefficient (PSC) was used to quantify the skewness of each length dataset of each marker, “PSC = 3 X ((mean − median)/standard deviation)”. PSC compares the distribution of the dataset with a normal distribution. The larger the coefficient value, the larger the distribution of the dataset differs from a normal distribution. When we looked at the distribution of the observed PSC values from all the microsatellite markers and CRC tissues, more than half of the observed PSC values from MSI-H CRCs were more than 1 or less than -1, whereas approximately 10% of the observed PSC values from MSS CRCs were more than 1 or less than -1 (S1 Fig). Based on such a finding, we defined a marker as unstable If the PSC value was more than 1 or less than -1. When over a certain fraction of all valid markers were unstable, the tumor sample was finally called MSI-H.

### Statistical analysis

The statistical analysis was performed with SPSS software for Windows, vesion 25.0 (IBM, Chicago, IL, USA). McNemar’s test was used to compare the sensitivities and specificities of mSILICO and mSINGS.

## Results

Based on our observations that in capillary electrophoresis of fluorochrome-labeled microsatellite sequences, MSI-H tumors display a more skewed distribution of the PCR products than those of MSS tumors, we developed a computational method for NGS-based determination of MSI, called mSILICO, which determines the skewness in the distribution of read lengths on each microsatellite locus and calls instability of each locus based on PSC>1 or <-1. To evaluate mSILICO’s performance, we compared its performance with that of mSINGS in the detection of MSI in CRCs. Immunohistochemistry for MMR proteins, including MLH1, MSH2, MSH6, and PMS2, was performed to confirm MSI statuses which were determined in CRC tissues by NCI’s five-marker panel (BAT25, BAT26, D2S123, D5S346, and D17S250). There was no discrepancy between expression status of MMR proteins and MSI status determined by NCI’s panel.

### Comparison of sensitivity and specificity in the detection of MSI according to the number of MSI markers

**Fig 1:**
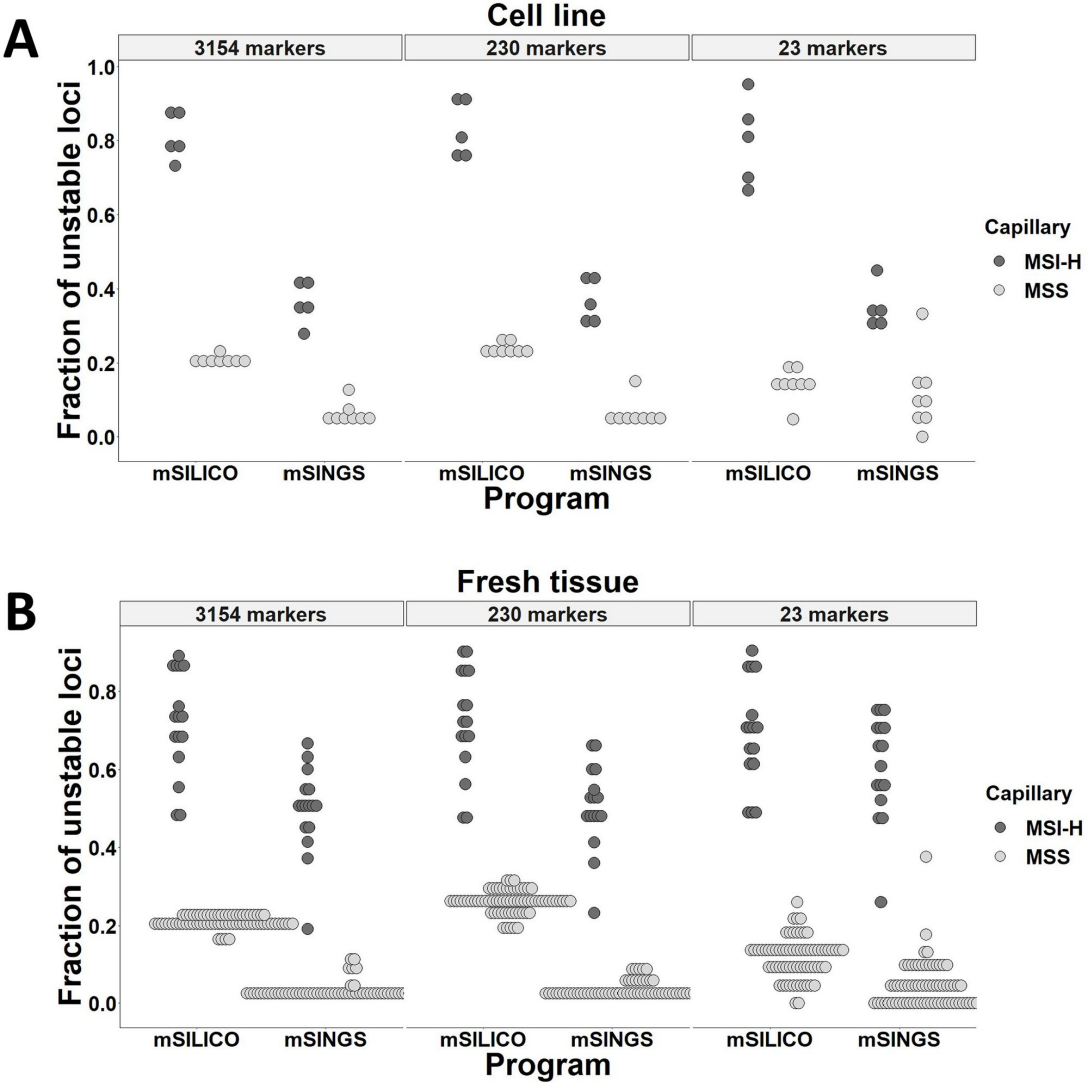
Comparison of the performances of mSILICO and mSINGS in the detection of microsatellite instability. A total of 3,154, 230, and 23 markers were analyzed for their instabilities in colorectal cancer cell lines (A) and fresh colorectal cancer tissues (B).

Eighty-four paired fresh CRC tissues of which MSI status had been determined by PCR-MSI were recruited. Sixteen cases were positive for MSI, and the others were negative for MSI. Thirteen cell lines, including five MSI-H cell lines, were also included for the analysis of MSI on the NGS platform. First, the MSI detection performances of mSILICO and mSINGS were compared in 13 CRC cell lines (Fig 1A) and 84 fresh CRC tissues (Fig 1B) for 3,154 microsatellite markers. Regardless of the type of computational method, MSI-H and MSS CRC tumors and cell lines were distinctly distributed along the axis representing the fraction of unstable markers. Cell lines tended to be more widely separated in the fraction of unstable markers with application of mSILICO than with that of mSINGS.

To evaluate whether the number of interrogated microsatellite markers could affect the sensitivity and specificity in the detection of MSI, two additional panels of microsatellite loci that included 230 and 23 loci were assessed for their performance in the detection of MSI by mSINGS and mSILICO (Fig 1A and 1B). For the 230-marker panel, MSI-H and MSS tumors were distinctly distributed regardless of the computational method. However, for the 23-marker panel, partial overlap was seen between MSI-H and MSS fresh CRC tissues and between MSI-H and MSS CRC cell lines with application of mSINGS. One (12.5%) of eight MSS CRC cell lines was misdiagnosed into MSI-H and one (1.5%) of 68 MSS CRC tissues was misplaced into MSI-H. In contrast, no overlap was found between MSI-H and MSS CRC tissues or between MSI-H and MSS CRC cell lines with the application of mSILICO.

For fresh tissue and cell line samples, a fraction of ≥0.4 (≥40% unstable loci) and a fraction of ≥0.2 (≥20% unstable loci) were used as cut-offs for MSI-H in mSILICO and mSINGS computational methods, respectively. These cut-off values were determined empirically. When the MSI-PCR results were used as the reference, the 3,154-, 230-, and 23-marker panels showed a sensitivity of 100% and a specificity of 100% in both cell lines and fresh tissues with the application of mSILICO. However, with the application of mSINGS, the 3,154-, 230-, and 23-marker panels had sensitivities of 100% and specificities of 100%, 100%, 87.5%, respectively, in cell lines and sensitivities of 93.8%, 100%, and 100%, respectively, and specificities of 100%, 100%, and 98.5%, respectively, in fresh tissues (Table 1).

**Table 1:** Performance of computational methods (mSINGS and mSILICO) and microsatellite marker panels (3,154, 230 and 23 markers) in CRC cell lines and fresh tissue samples.

			Cell line	Fresh tissue
3,154	mSINGS	Sensitivity	100%	93.8%
		Specificity	100%	100%
		Accuracy	100%	98.8%
	mSILICO	Sensitivity	100%	100%
		Specificity	100%	100%
		Accuracy	100%	100%
230	mSINGS	Sensitivity	100%	100%
		Specificity	100%	100%
		Accuracy	100%	100%
	mSILICO	Sensitivity	100%	100%
		Specificity	100%	100%
		Accuracy	100%	100%
23	mSINGS	Sensitivity	100%	100%
		Specificity	87.5%	98.5%
		Accuracy	92.3%	98.8%
	mSILICO	Sensitivity	100%	100%
		Specificity	100%	100%
		Accuracy	100%	100%

No difference in sensitivities and specificities of 23-, 230-, 3,154-marker panels between mSINGS and mSILICO (by McNemar test)

### The performance of mSILICO in FFPE tissue samples

**Fig 2:**
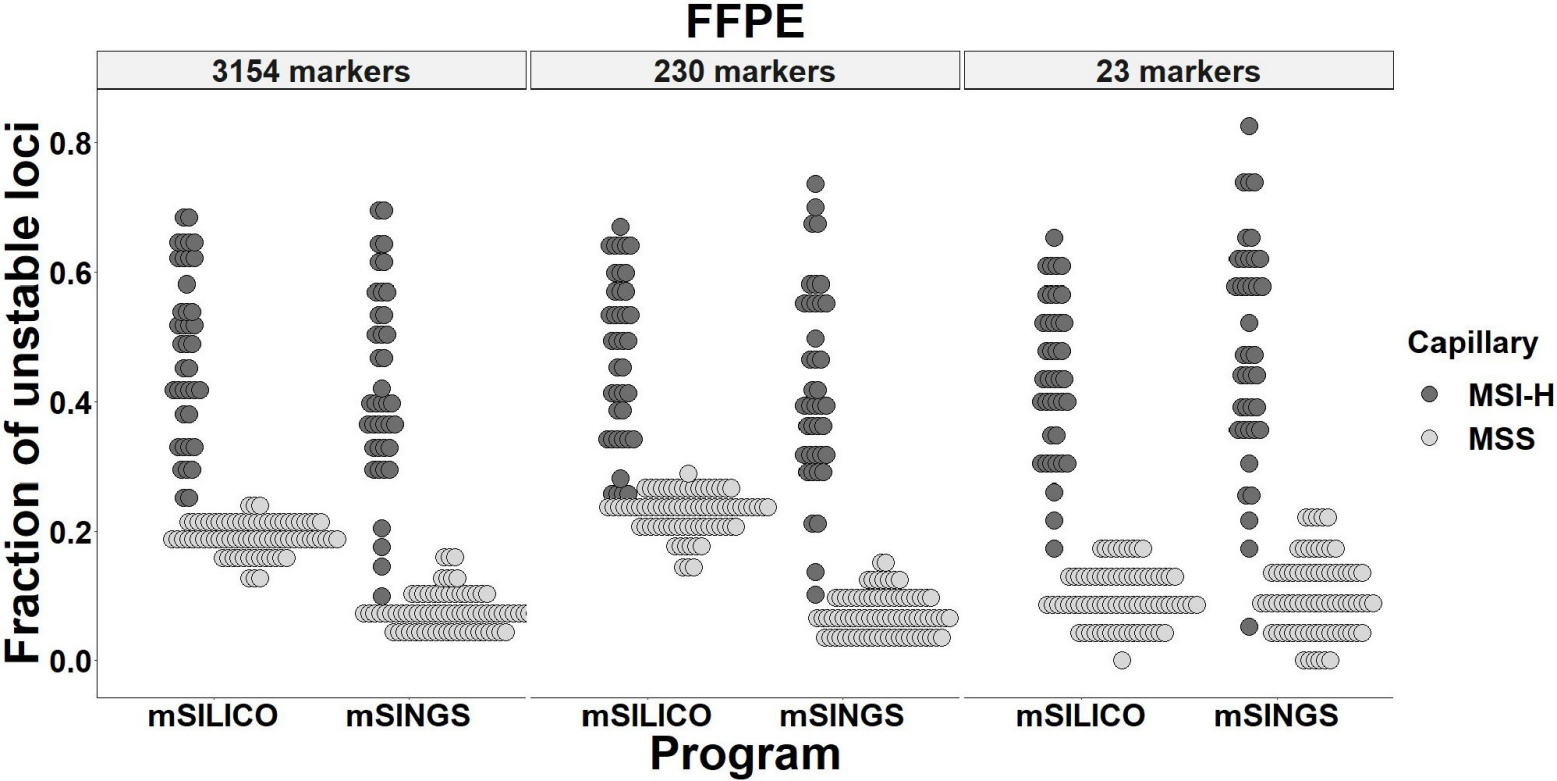
mSILICO and mSINGS were evaluated for their performance in the detection of microsatellite instability in formalin-fixed paraffin-embedded (FFPE) tissues of colorectal cancers using three different marker panels.

Next, to identify whether the mSILICO computational method works in FFPE tissue samples as well as in fresh tissue samples or cell lines, two computational methods coupled with three different marker panels were compared to determine their performance in the detection of MSI with FFPE tissue samples. FFPE tissue samples of 40 MSI-H and 77 MSS CRCs whose MSI status using MSI-PCR had been previously determined were retrieved and subjected to MSI-NGS. Regardless of the number of panel markers, partial overlap between MSI-H and MSS tumors was seen in mSINGS (Fig 2). Although no distinct separation between MSI-H and MSS tumors was noted in the 3,154-, 230-, and 23-marker panels with application of mSILICO, mSILICO showed a lower degree of overlap between MSI-H and MSS tumors in the 3,154- and 23-marker panels compared with mSINGS. With a cut-off value of 0.25, the 3,154-, 230- and 23-marker panels had a sensitivity of 100%, 100%, and 95%, respectively, and a specificity of 100%, 77.2% and 100%, respectively, with application of mSILICO. With the application of mSINGS and a cut-off value of 0.20, the 3,154-, 230- and 23-marker panels had sensitivities of 92.5%, 95%, and 95%, respectively, and specificities of 100%, 100%, and 93.7%, respectively (Table 2).

**Table 2:** Performance of computational methods (mSINGS and mSILICO) and microsatellite marker panels (3,154, 230 and 23 markers) in formalin-fixed paraffin-embedded (FFPE) tissue samples.

			FFPE
3,154	mSINGS	Sensitivity	92.5%
		Specificity	100%
		Accuracy	97.5%
	mSILICO	Sensitivity	100%
		Specificity	100%
		Accuracy	100%
230	mSINGS	Sensitivity	95.0%
		Specificity	100%
		Accuracy	98.3%
	mSILICO	Sensitivity	100%
		Specificity	77.2%
		Accuracy	84.9%
23	mSINGS	Sensitivity	95.0%
		Specificity	93.7%
		Accuracy	94.1%
	mSILICO	Sensitivity	95.0%
		Specificity	100%
		Accuracy	98.3%

A significant difference in sensitivity and specificity of 230-marker panel but no differences in 23-marker panel and 3,154-maker panel between mSILICO and mSINGS (by McNemar test)

## Discussion

In the present study, we developed a new computational method, mSILICO, which utilizes the skewness in the distribution of the read lengths at each microsatellite locus. When compared with mSINGS, mSILICO showed comparable results in the sensitivity and specificity of MSI detection in both fresh tissue or cell line samples and FFPE tissue samples. In our study, the performance of the 23-marker panel was similar to that of the 3,154-marker panel in the detection of MSI regardless of whether genomic DNA samples were extracted from fresh tissues or formalin-fixed tissues. Such a small panel can be added to existing targeted exome panels, reinforcing the performance of MSI analysis. Although targeted exome sequencing of ≥275 genes (encompassing 757,787 bp of the genome) was demonstrated to differentiate MSI-H from MSS CRCs at a sensitivity and specificity of ≥99% using tumor mutational load [21, 22], information regarding a mutational load obtained from the smaller genomic space sampled by gene panels, is unlikely to clearly differentiate MSI-H CRCs from MSS CRCs, where a panel of 23 microsatellite markers could help to diagnose MSI accurately.

When the performance of mSILICO and mSINGS for MSI-NGS was compared among cell lines, fresh frozen and FFPE tissue samples, the determination power was the lowest in the FFPE group regardless of the computational method. For the cell lines and fresh tissue samples, the MSI-H and MSS groups were clearly distinguished in mSILICO, regardless of the number of panel markers. Rather, in the control method, mSINGS, false-negative or false-positive results occur in sample types of cell lines and fresh tissue. For FFPE samples with the use of 23 or 3,154 markers, mSILICO tended to show higher accuracy than mSINGS, although mSILICO exhibited lower specificity in the 230-marker panel than mSINGS. Another point that favors mSILICO over mSINGS is that while mSINGS needs MSS samples (n = 10–20) to set the baseline, mSILICO can detect MSI without recruiting MSS sample data. Additionally, the running time of mSINGS for determination of MSI status was longer than that of mSILICO (S2 Fig). However, the cut-off value of the unstable fraction was constant in mSINGS regardless of whether tissue samples were fresh or formalin-fixed, whereas mSILICO showed different cut-off values for determining MSI-H depending on whether the tissue sample was fresh or fixed. In our mSILICO results, the cut-off value for determination of MSI differed between FFPE tissues and cell lines/fresh tissues. For fresh tissues, the cut-off value could be set at 0.4 regardless of panel size (23, 230, and 3,154 markers), whereas for FFPE tissues, the cut-off value could be set at 0.25. Further optimization of the cut-off value should be performed with a large-scale sample of CRC tissue samples.

In DNA samples obtained from cell lines or fresh frozen tissues, the fraction value of unstable loci was clearly separated between MSI-H tumors and MSS tumors, whereas in DNA samples extracted from FFPE tissues, the fraction of unstable loci was distributed with partial overlap between MSI-H tumors and MSS tumors. The tendency of incomplete separation in relation to the use of genomic DNA from FFPE tissue was also found in the control computational method, mSINGS, and in other MSI-NGS studies using mSINGS [16], which suggests that complete differentiation between MSI-H and MSS tumors might be difficult to accomplish with current NGS and analytical techniques. For such a tumor with borderline fraction value, information on tumor mutational load might help to differentiate MSI-H from MSS tumors.

For MSI-H tumors, the fraction of unstable loci tended to be decreased and widely distributed in FFPE tissues compared with that of fresh tissues regardless of the computational method and the number of panel markers. For MSS tumors, the fraction of unstable loci tended to be more widely distributed in FFPE tissues than in fresh tissues, regardless of the computational method and the number of panel markers. The reason why the fraction of unstable loci was more widely distributed in FFPE tissues than in fresh tissues might be related to sequencing artifacts, which can be caused by damage to DNA due to fixation with formalin, sample storage at room temperature and DNA extraction procedures. Of FFPE-associated sequencing artifacts, including G-C bias, increased base-error rate, decreased proportion of properly paired read alignment, strand-split artifact reads, and aberrant detection of copy number gain or loss [23–25], it is unclear which ones are involved in wide distribution of the fraction of unstable loci in association with formalin fixation. When we analyzed the mean number of reads along the markers in each tumor or cell line and the mean number of reads along the samples in each marker, FFPE tissues showed much wider variation in the mean number of reads than fresh tissues or cell lines (S3 Fig). Each marker also exhibited wider variation in the number of reads in FFPE tissue samples than in fresh tissues or cell lines. A lower number of reads might lead to underrepresentation of the alleles, which might generate a tendency toward a decreasing absolute value of PSC in each marker, finally resulting in a decreased fraction of unstable loci in FFPE tissue samples.

The limitation of our study was that we did not analyze the performance of mSILICO and marker panels in other tissue types of cancers in which MSI occurs as frequently as in CRCs. Thus, we do not know whether mSILICO and marker panels work in gastric cancers or endometrial cancers as well as in CRCs. The baseline microsatellite status for this dataset was determined using MSI-PCR, which was performed with Bethesda’s five- marker panel. Bethesda’s five-marker panel is known to be inferior to pentaplex PCR. Thus, a few missing MSI tumors might be included in MSS tumors. However, mSILICO and mSINGS did not find any MSI-H tumors in MSS tumors.

In summary, we developed a computational method that can differentiate MSI-H tumors from MSS tumors without paired normal tissue samples and even baseline normal samples. A small panel of 23 markers can be added to existing gene panels for targeted exome sequencing, which are not able to diagnose MSS tumors with mutational load alone.

## Supporting information

S1 Table List of genes. (DOCX)

S2 Table Twenty-three microsatellite markers. (DOCX)

S1 Fig Distributions of Pearson’s skewness coefficient values in microsatellite instability-high and microsatellite-stable colorectal cancers. (TIF)

S2 Fig The two programs differ in the time it takes because mSINGS requires several more steps. (TIF)

S3 Fig The number of reads was obtained in 23 microsatellite markers, and the mean number of reads (A) and standard deviation (SD) were compared among colorectal cancer cell lines, formalin-fixed, paraffin-embedded (FFPE), and fresh colorectal cancer tissues. (TIF)
