# Supplementary material for: Targeted next-generation sequencing-based detection of microsatellite instability in colorectal carcinomas
Source: PLoS One. 2021 Feb 1;16(2):e0246356. doi: 10.1371/journal.pone.0246356 (PMC7850495; doi:10.1371/journal.pone.0246356)
Supplement: S1 Table — (DOCX) [file pone.0246356.s001.docx]

S1 Table. List of genes.

| 1 | ACVR1B | 18 | ERBB2 | 35 | NRAS |
| --- | --- | --- | --- | --- | --- |
| 2 | ACVR2A | 19 | ERBB3 | 36 | PDGFRA |
| 3 | ALK | 20 | ERBB4 | 37 | PIK3CA |
| 4 | AMER1 | 21 | FBXW7 | 38 | PIK3R1 |
| 5 | APC | 22 | FZD10 | 39 | PTEN |
| 6 | ARID1A | 23 | HRAS | 40 | RNF43 |
| 7 | ATM | 24 | IDH1 | 41 | SETDB1 |
| 8 | BRCA1 | 25 | IDH2 | 42 | SMAD2 |
| 9 | BRCA2 | 26 | IGF1R | 43 | SMAD3 |
| 10 | AXIN2 | 27 | IGF2 | 44 | SMAD4 |
| 11 | BRAF | 28 | IRS2 | 45 | SOX9 |
| 12 | CTNNB1 | 29 | KRAS | 46 | SRC |
| 13 | DKK1 | 30 | KIT | 47 | TCF7L2 |
| 14 | DKK2 | 31 | LRP5 | 48 | TGFBR1 |
| 15 | DKK3 | 32 | MTOR | 49 | TGFBR2 |
| 16 | DKK4 | 33 | MYC | 50 | TP53 |
| 17 | EGFR | 34 | MYCN |  |  |
