# Supplementary material for: Targeted next-generation sequencing-based detection of microsatellite instability in colorectal carcinomas
Source: PLoS One. 2021 Feb 1;16(2):e0246356. doi: 10.1371/journal.pone.0246356 (PMC7850495; doi:10.1371/journal.pone.0246356)
Supplement: S2 Table — (DOCX) [file pone.0246356.s002.docx]

S2 Table. Twenty-three microsatellite markers.

|  | Chromosome | Marker |
| --- | --- | --- |
| 1 | 1 | RNF19B |
| 2 | 1 | MTMR11 |
| 3 | 1 | MTR |
| 4 | 2 | BAT26 |
| 5 | 2 | ACVR2A |
| 6 | 3 | TGFBR2 |
| 7 | 3 | FOXP1 |
| 8 | 3 | KIAA2018 |
| 9 | 3 | MYLK |
| 10 | 4 | KIT |
| 11 | 4 | SEC31A_Exonic_MSI |
| 12 | 5 | D5S346 |
| 13 | 6 | ADTRP |
| 14 | 7 | GTF2IP1_LOC100093631 |
| 15 | 11 | CADM1 |
| 16 | 11 | PUS3 |
| 17 | 14 | C14orf169 |
| 18 | 17 | CRK |
| 19 | 17 | TP53 |
| 20 | 17 | RNF43 |
| 21 | 18 | C18orf56 |
| 22 | 19 | RFX1 |
| 23 | X | BEX5 |
