# Supplementary figures and images for: Targeted next-generation sequencing-based detection of microsatellite instability in colorectal carcinomas
Source: PLoS One. 2021 Feb 1;16(2):e0246356. doi: 10.1371/journal.pone.0246356 (PMC7850495; doi:10.1371/journal.pone.0246356)

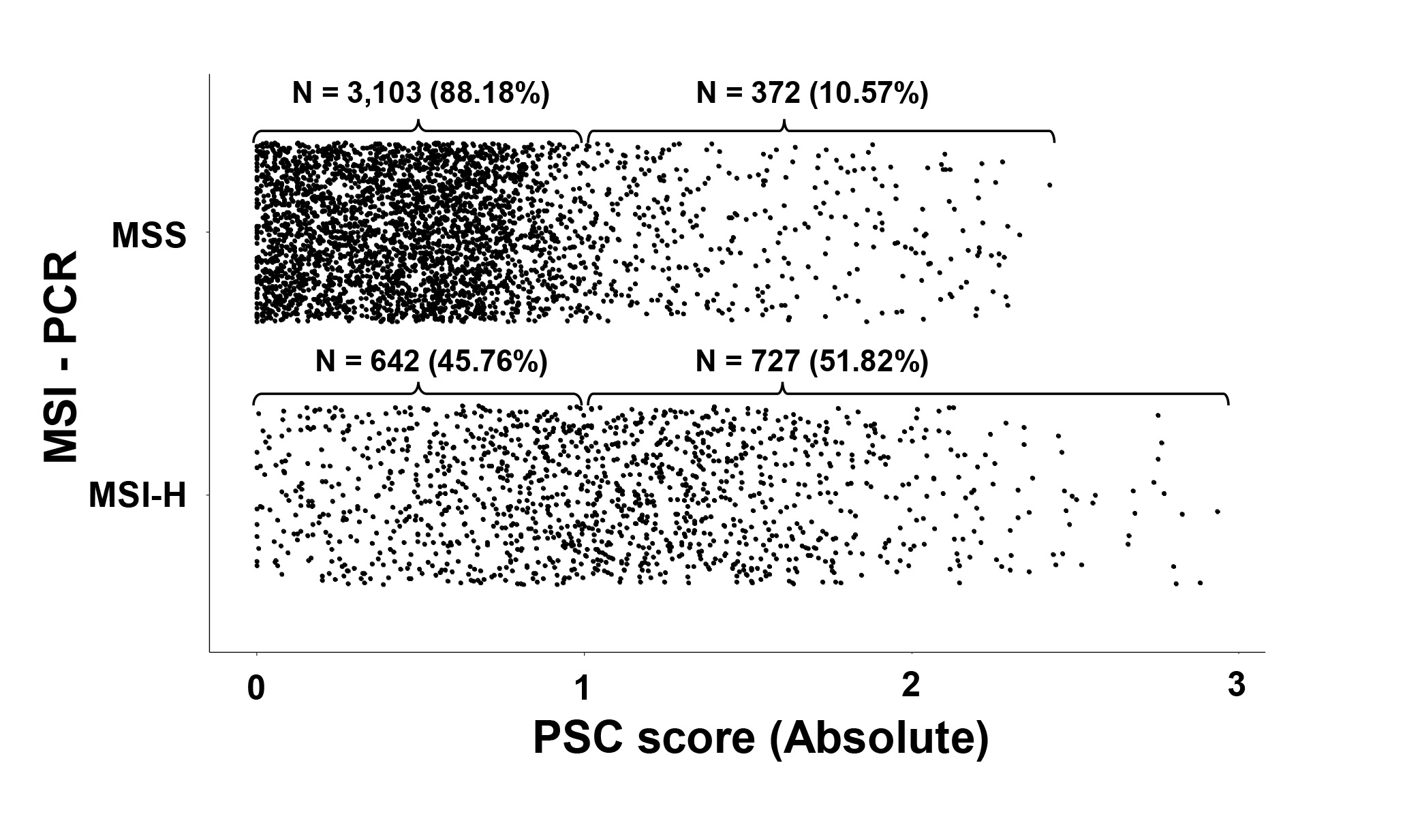

Supplement: S1 Fig — (TIF) [file pone.0246356.s003.tif]

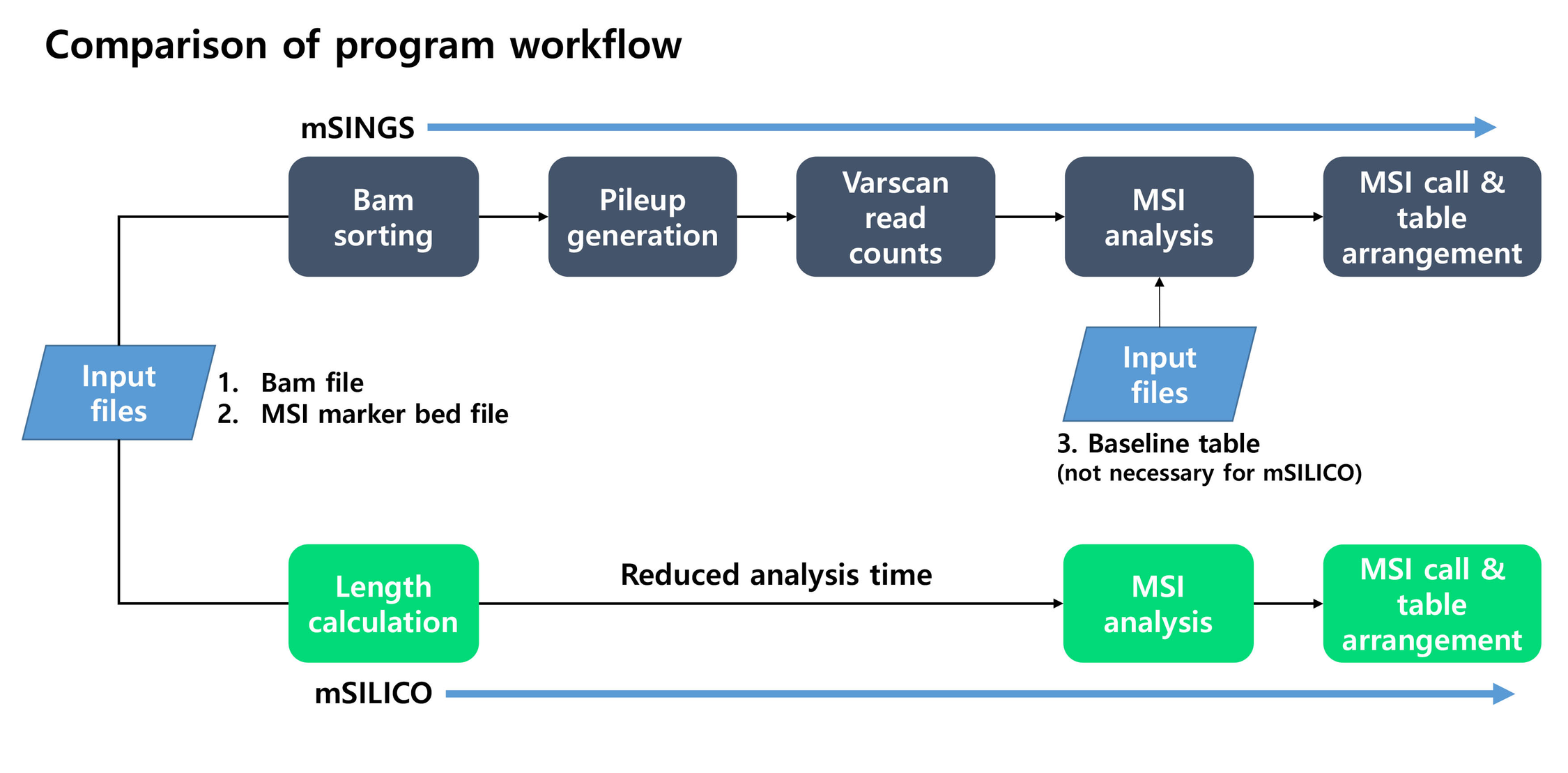

Supplement: S2 Fig — (TIF) [file pone.0246356.s004.tif]

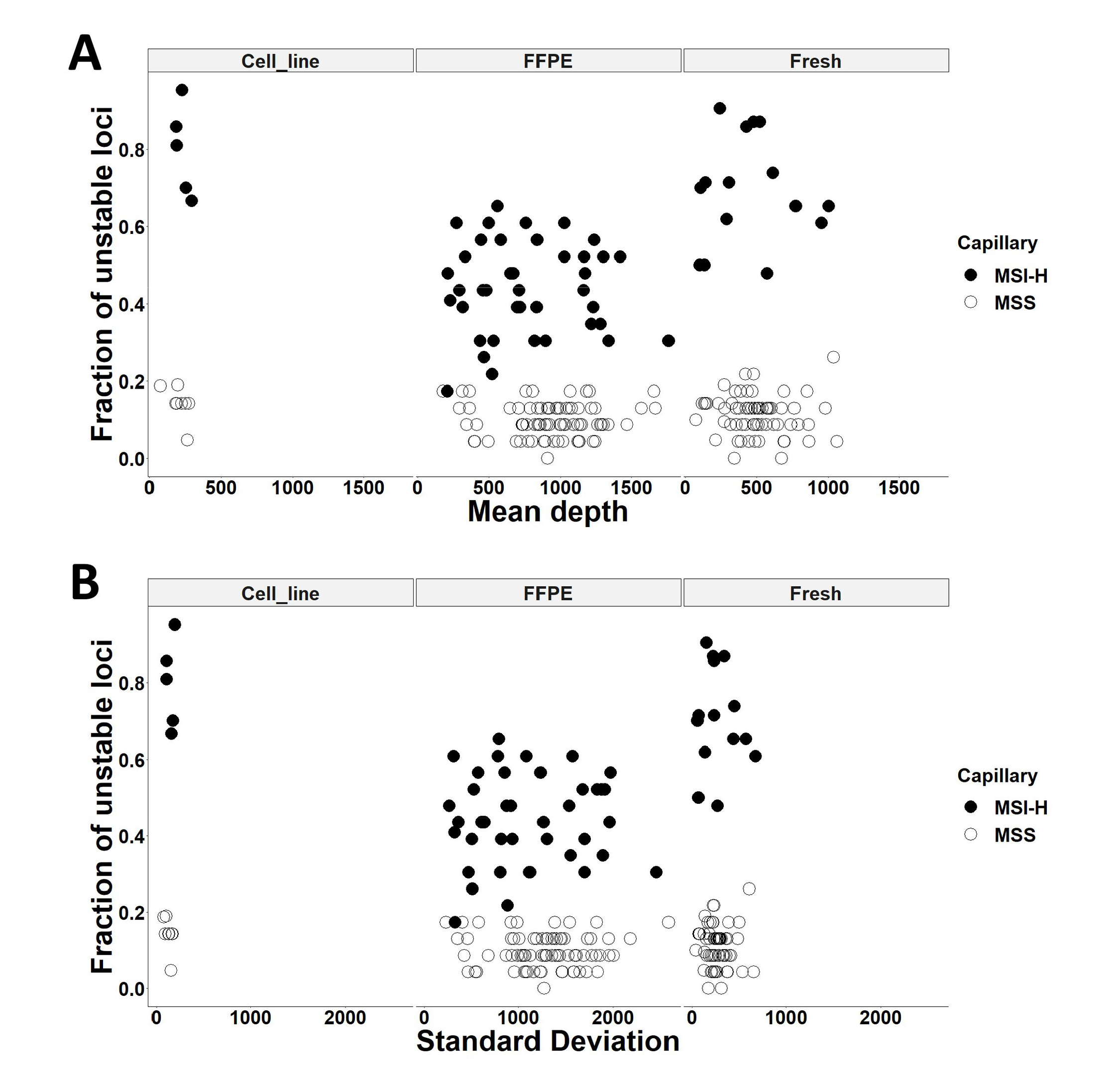

Supplement: S3 Fig — (TIF) [file pone.0246356.s005.tif]
